# Enhanced wound repair ability of arginine-chitosan nanocomposite membrane through the antimicrobial peptides-loaded polydopamine-modified graphene oxide

**DOI:** 10.1186/s13036-021-00268-3

**Published:** 2021-05-22

**Authors:** Chuan Fu, Zhiping Qi, Chengliang Zhao, Weijian Kong, Hongru Li, Wenlai Guo, Xiaoyu Yang

**Affiliations:** 1 Department of Orthopaedic Surgery, The Second Hospital of Jilin University, Changchun, 130021 China; 2 Department of Spinal Surgery, The Affiliated Hospital of Qingdao University, Qingdao, 266000 China

**Keywords:** Chitosan, Graphene oxide, Antimicrobial peptides, Arginine, Wound dressing

## Abstract

Skin wound healing is a complicated and lengthy process, which is influenced by multiple factors and need a suitable cellular micro-environment. For skin wound, wound dressings remain a cornerstone of dermatologic therapy at present. The dressing material can create an effective protective environment for the wound, and the interactions between the dressing and the wound has a great impact on the wound healing efficiency. An ideal wound dressing materials should have good biocompatibility, moisturizing property, antibacterial property and mechanical strength, and can effectively prevent wound infection and promote wound healing. In this study, in order to design wound dressing materials endowed with excellent antibacterial and tissue repair properties, we attempted to load antimicrobial peptides onto dopmine-modified graphene oxide (PDA@GO) using lysozyme (ly) as a model drug. Then, functionalized GO was used to the surface modification of arginine-modified chitosan (CS-Arg) membrane. To evaluate the potential of the prepared nanocomposite membrane in wound dressing application, the surface morphology, hydrophilic, mechanical properties, antimicrobial activity, and cytocompatibility of the resulting nanocomposite membrane were analyzed. The results revealed that prepared nanocomposite membrane exhibited excellent hydrophilic, mechanical strength and antimicrobial activity, which can effectively promote cell growth and adhesion. In particular, using PDA@GO as drug carrier can effectively maintain the activity of antimicrobial peptides, and can maximize the antibacterial properties of the nanocomposite membrane. Finally, we used rat full-thickness wound models to observe wound healing, and the surface interactions between the prepared nanocomposite membrane and the wound. The results indicated that nanocomposite membrane can obviously accelerated wound closure, and the wounds showed reduced inflammation, improved angiogenesis and accelerated re-epithelialization. Therefore, incorporation of antimicrobial peptides-functionalize graphene oxide (ly-PDA@GO) into CS-Arg membrane was a viable strategy for fabricating excellent wound dressing. Together, this study not only prepared a wound dressing with excellent tissue repair ability, but also provided a novel idea for the development of graphene oxide-based antibacterial dressing.

**Supplementary Information:**

The online version contains supplementary material available at 10.1186/s13036-021-00268-3.

## Introduction

As the largest organ of the human body, skin plays an important role in the balance of the internal environment and the protection against microbial invasion [1]. Skin wound caused by burn, scalding, laceration, and surgery is one of the most important health problems in the whole world that can occurred in many parts of the body. In skin wound treatment, there is a great demand for wound dressing materials that can speed up wound healing and keep hemostasis, moist environment at the wound, prevent from infection [2]. Nowadays, gauze is the most commonly used dressing in clinic, but it have many shortcomings, including frequent replacement, tissue adhesion and the lack biological activity. In recent years, skin tissue engineering shows great potential in developing wound dressing for wound treatment. In order to prepare an ideal dressing, this biomaterials should have excellent biocompatibility, tissue repair ability and the capacity to provide defense against bacterial infection. Researchers have been focused on producing various wound dressing materials to promote skin tissue regeneration, such as gelatin, chitosan, collagen, silk fibroin and synthetic polymers [3–7].

Chitosan (CS), a natural alkaline polysaccharide with an excellent biocompatibility, have been reported to be safe, antibacterial, immune modulator, and promotes wound healing in vivo [8–10]. At present, CS and its composites are widely investigated for skin wound repair. The N-acetylglucosamine units in CS are much similar to the glycosaminoglycan in natural ECM, which can attract growth factors and biological proteins to participate in tissue regeneration. However, the low mechanical strength of CS is unfavourable for morphological and structural changes during skin healing, and CS alone possesses limited antimicrobial ability, which has restricted its regenerative stimulation of skin wound. Fortunately, the presence of amino and hydroxyl groups on the surface of CS makes it easier to be modified. To increase the cytocompatibility of CS materials, many experimental and theoretical studies have been used to modifying the CS. Particularly, in recent year, the introduction of amino acid moieties to the CS generates some interesting synergistic characteristics for expanding the application scope of CS, such as l-asparagine, l-arginine or l-lysine [11–13]. Arginine (Arg) is considered as one important amino acids in young mammals, and it can regulates cell division, immune function, and hormone release [14, 15]. Previous studies have demonstrated Arg can enhanced the mesenchymal stem cell adhesion and proliferation [16]. Shi et al. have demonstrated the Arg also possess the capacity to improve the collagen deposition, which is fundamental for the skin wound healing process [17]. More importantly, the process of Arg metabolism generates a essential nitrogen-containing compounds (nitric oxide, NO), which has an antibacterial function and is also critical to the collagen accumulation of newborn tissue [18]. Based on the physiological roles of this amino acid, the Arg grafting can also be used as a strategy to improve the bioactivity of the CS materials.

During skin wound treatment, bacterial infection is an important factor affecting wound healing. Bacterial infection can lead to an increase in exudate from the wound site, and inhibit the formation of granulation tissue, thereby inhibiting wound healing. In order to prevent bacterial infection, antibiotics such as penicillin and methicillin are often used to improve the antibacterial properties of wound dressings. However, it is necessary to find effective alternatives for broad-spectrum antibiotics due to the abuse of antibiotics and the emergence of antibioticresistant bacteria [19]. In recent years, the antimicrobial peptides including cathelicidins, defensins, lysozyme and lactoferrin, which have shown great application value in the treatment of wound infection [20–23]. Those antimicrobial peptides are ubiquitous in all eukaryotic organisms and are an essential element of the immunesystem, which are consideredmuch safer than conventional antibiotic. However, the half-life of antimicrobial peptides is short, and they are easy to deactivate when applied in vivo alone. Furthermore, antimicrobial peptides readily bind to other proteins, which lead to their inactivation. Therefore, the therapeutic efcacy of antimicrobial peptides often depends on a efficient delivery carrier. Graphene oxide (GO), a derivative of graphene with a typical quasi-two-dimensional (2D) spatial structure, is recognized among the most exciting carbon nanomaterials [24–26]. GO has a large specific surface area, and a large number of reactive oxygen-containing functional groups, such as epoxy groups, hydroxyl groups, and carboxylic acid groups [27].These features can enhance the binding affinity of GO with growth factor or drug via physical adsorption and electrostatic interactions. Hence, GO can selected as a drug carrier candidate for biomaterials functionalization, and play significant role to overcome the challenges in drug therapy [28]. However, many studies have found GO can significantly changes the secondary and tertiary structure of protein polypeptide drugs, thereby inhibiting the activity of protein polypeptide drugs [29].. The loss of protein polypeptide activity will seriously interfere with the metabolism and other functions of the biological system, leading to toxic reactions [30]. Bai et al. used GO to adsorb lysozyme and found that the activity of lysozyme was seriously inhibited [29]. Therefore, GO needs to be pretreated before it can be used as a drug carrier. Dopamine (DA) is a neurotransmitter that can form a well-adhering poly-dopamine (PDA) layer on the surface of various substances in an alkaline environment [31]. Furthermore, the functional groups in dopamine (such as catechols, amines, and imines) can serve as active sites for covalent modification of ideal molecules. It is report that PDA can be used as the reducing and capping reagent of GO, which can effective improve the stability and dispersity of GO and reduce its destructive effect on protein polypeptide activity [32].

At present, many studies have used dopamine-coated GO (PDA@GO) as the carrier of protein drugs to treat various diseases such as tumor, and achieved good therapeutic effects [33, 34]. But there is rare investigation about the effect of PDA@GO loaded with antimicrobial peptides on the biological properties of wound dressing materials. Based on these considerations, in this study, lysozyme (ly) was selected as a model antimicrobial peptide drug because it is readily available and its bioactivity has been reported in many previous studies. Subsequently, Ly-functionalized PDA@GO (ly-PDA@GO) was added in CS-Arg nanocomposite membrane to improve the antibacterial properties and biocompatibility of the wound dressing materials. Our aim was to investigate whether this nanocomposite membrane could be used as an ideal dressing materials for skin wound treatment. The physicochemical, mechanical, antibacterial properties and cell compatibility of nanocomposite membrane are checked considering the biomedical uses of resultant nanocomposite membrane. This study further showed the effects of functionalized GO in antimicrobial peptides delivery, and the potential of surface modified CS-based dressing materials for applications of the skin wound repair.

## Materials and methods

### Materials

Chitosan (degree of deacetylation = 98%) was purchased from purchased from VETEC (Shanghai, China). L-Arginine (Arg), N-hydroxysuccinimide (NHS) were purchased from Aladdin Industrial Corporation (Shanghai, China). 1-(3-Dimethylaminopropyl)-3-ethylcarbodiimide (EDC) was purchased from Shanghai Jonln Industrial Corporation (Shanghai, China). 3-Hydroxytyramine (Dopamine) hydrochloride was purchased from Adamas Reagent Co. Ltd. Lysozyme were purchased from GL Biochem (Shanghai) Co., Ltd. GO was purchased from Chengdu Organic Chemicals Co. Ltd., China (thickness: 0.55–1.2 nm diameter: 0.5–3 μm). The reagents for cell experiments were purchased from Gibco (USA).

### Synthesis and characterization of CS-Arg

According to our previous work [35], the l-arginine grafted chitosan (CS-Arg) was prepared by amidation of the primary amine groups present in CS glucosamine (GlcN) units by using EDC/NHS as coupling agents. Briefly, chitosan (0.4 g) was dissolved in 1% acetic acid (20 ml) to form homogeneous aqueous solution at ambient temperature. Subsequently, NHS was dissolved in the above solution (0.55 mol/mol EDC), under intense magnetic stirring. EDC was then added to the reaction (1.5 mol/mol l-arginine). Finally, l-arginine were dissolved in the above solultions and subsequent incubation with stirring for 24 h at room temperature. The resultant product was purified using a dialysis tube (200 MWCO) against distilled water for 3 days. The purified CS-Arg polymer was finally recovered by freeze-drying for 24 h. Fourier-transform infrared spectroscopy (FT-IR, VERTEX 70; Bruker) was used to examine the chemical properties of CS-Arg.

### Preparation and characterizationof GO and PDA@GO

The prepare method of PDA@GO was followed as previously described [36]. Briefly, GO nanoparticles was first dispersed in 100 ml Tris-Buffer solution (10 mM, pH = 8.5), followed by continuous ultrasound for 30 min. Then, dopamine hydrochloride (2 mg/mL) was added to the above GO solution and stirred at room temperature for 24 h. Finally, PDA@GO was centrifuged and repeatedly washed with deionized water and ethanol to remove residual PDA. The prepared PDA@GO is freeze-dried and stored for the following experiments.

### The adsorption of lysozyme and fabrication of ly-PDA@GO/CS-Arg membrane

Six milligram GO and PDA@GO were placed in centrifuge tubes respectively, and then 2 mL lysozyme solution (2 mg/mL) was added. Subsequently, the above mixed solution was fully shaken at room temperature for 2 h. The prepared ly-GO and ly-PDA@GO was centrifuged and collected. All supernatants were collected, and re-frozen and thawed. BCA kit was used to measure the adsorption amount of lysozyme. In order to fabricated ly-PDA@GO/CS-Arg nanocomposite membrane, CS-Arg powder is dissolved in 0.5% (V/V) acetic acid solution, and CS-Arg solution then was spread uniformly on glass plate. After drying for 48 h, dried CS-Arg membrane was cross-linked and wasted by deionized water. To immobilize the ly-PDA@GO on the CS-Arg nanocomposite membrane surfaces, CS-Arg membrane were immersed in ly-PDA@GO solution and shaking continuously for 12 h. Finally, the prepared membranes were washed with deionized water to remove any unbound ly-PDA@GO and dried in the air for subsequent experiments. Meanwhile, pure CS, CS-Arg and ly-GO/CS-Arg nanocomposite membrane were also prepared under the same conditions.

### Characterization of the ly-PDA@GO/CS-Arg nanocomposite membranes

The surface morphology of different nanocomposite membranes were observed by scanning electron microscope (SEM, XL 30 ESEM-FEG, FEI) and multimode scanning probe atomic force microscope (AFM, Veeco Instruments). The nanocomposite membranes were cut into rectangular strips of 10 × 30 × 0.05 mm in size. A universal mechanical tester (Instron 1121, UK) was used to test the mechanical properties of different nanocomposite membrane. In order to detect the hydrophilicity of different nanocomposite membrane surfaces, hydrostatic contact angles were measured by a contact angle system (VCA 2000, AST).

### Antibacterial testing

Antibacterial testing was conducted using *Staphylococcus aureus* (*S. aureus*) and Escherichia coil (*E. coli*). Briefly, the original bacterial solution (4.0 × 10^4^ ml^− 1^) was cultured at 37 °C for 2 h. Subsequently, different nanocomposite membrane were added to above bacterial solution. Then, the above solution was incubated at 37 °C for 10 h. Finally, 0.1 ml of liquid was extracted from each group and transferred to a new 96-well plate. The OD was measured at 600 nm using the multifunction microplate scanner.

To better observe the bacteriostatic effect of different nanocomposite membrane, the live/dead assay was used to observed changes in bacteria after treatment with different nanocomposite membrane. Different nanocomposite membrane and bacterial suspension were co-cultured for 12 h at 37 °C. Subsequently, PI and Calcein were used to stain bacterial cells. Finally, a fluorescence microscope (TE2000-U; Nikon) was used to observed different samples.

### Cell adhesion and proliferation assay

Cell proliferation on the CS, CS-Arg, ly-GO/CS-Arg and ly-PDA@GO/CS-Arg nanocomposite membrane was assessed. The nanocomposite membrane were processed into discs with a diameter of 10 mm, treated by ultraviolet irradiation for 40 min, and placed in a 24-well plate. NH3T3 cells were then seeded on different nanocomposite membrane surfaces at a density of 2 × 10^4^ cells/wells and cultured at 37 °C, 5% CO_2_, and 95% humidity for 7 days. At each specific time point, CCK-8 assay was used to evaluate the effects of different materials on cell proliferation. Briefly, the medium was replaced by Cell Counting Kit-8 (CCK-8, Dojindo, Japan). The samples were then incubated at 37 °C for 2 h. Finally, the 100 μl medium was transferred to a new 96-well plate and the absorbance was measured at 450 nm with a multifunction microplate scanner.

For cell adhesion assessment, NH3T3 cells were cultured on different nanocomposite membrane. After 4 days of culture, the cells were fixed with 4% paraformaldehyde for 10 min and washed with PBS for three times. FITC and DAPI were subsequently used for cytoskeleton and nucleus staining, respectively. Finally, the cell morphology were observed under a fuorescence microscope.

### In vivo wound-healing assay

Adult female rats (10–12 weeks old, weighing 200–250 g) were used for bioactivity analysis of different materials. Rats were anesthetized with 2% pentobarbital sodium and then back hair was removed. A round full-thickness skin wound model on the back of rats (diameter 10 mm) was made by ophthalmic scissors. Then, various nanocomposite membrane (CS, CS-Arg, ly-GO/CS-Arg and ly-PDA@GO/CS-Arg) was applied to cover the skin wound of rats, and the wound dressing was changed every 2 days. The experimental animals were kept in single cages at standard environment (22 °C). Granular feed is provided regularly and each animal has free access to food and water. All experimental animals were provided by Animal Experimental Center of Jilin University (license No.: SCXK (Ji) 20,110,004) and randomly divided into 5 groups (*n* = 3 for each group). At 0, 5, 9 and 12 days after surgery, the wound was photographed with a digital camera, and the wound area was measured with the software Image J. The wound closure rate was calculated by the following formula:

$$ \mathrm{Wound}\ \mathrm{closure}\ \mathrm{rate}==\left[\left(\mathrm{A}0-\mathrm{At}\right)/\mathrm{A}0\right]\times 100\% $$

where A0 indicates the initial area of the wound (t = 0), and At indicates the wound area at the time of measurement.

### Histopathological assay

After 12 days of post-surgery, the rats were killed and the skin samples on the wound site were removed and carefully trimmed with a cutter. Then, the skin tissue was fixed with a 4% paraformaldehyde solution. After paraffin embedding, skin tissue was sliced into 4 um slices. Sections were then stained with Masson and H&E,and observed using a light microscope. Furthermore, in order to observe the collagen deposition at the wound, the tissue samples were stained with Sirius red-picric acid solution and observed with polarizing light microscope. The ratio of type I and type III collagen was quantitatively analyzed. Meanwhile, the wound sections were Immunofluorescence stained with VEGF to evaluate angiogenesis.

### Statistical analyses

All quantitative date were analyzed by one-way analysis of variance using Origin 8.0 software (Origin Lab Corporation, USA). Data are expressed as the mean ± SD. *P* value less than 0.05 was considered statistically significant in all analyses.

## Results and discussion

### Preparation of ly-PDA@GO

**Scheme 1:**
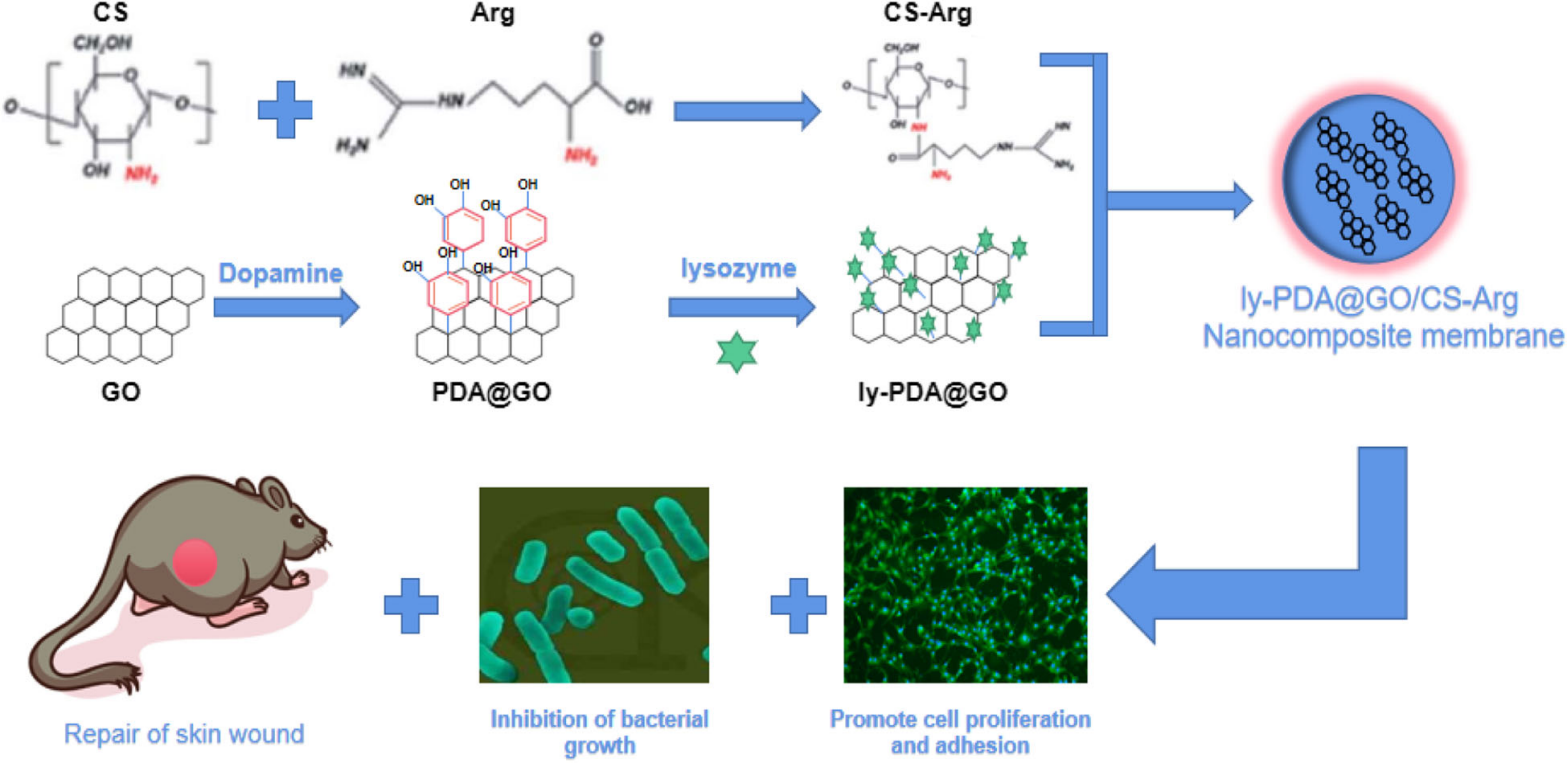
Schematic illustration of the preparation of the CS-Arg nanocomposite membrane modifed with ly-PDA@GO coating and its application for wound treatment

**Fig. 1:**
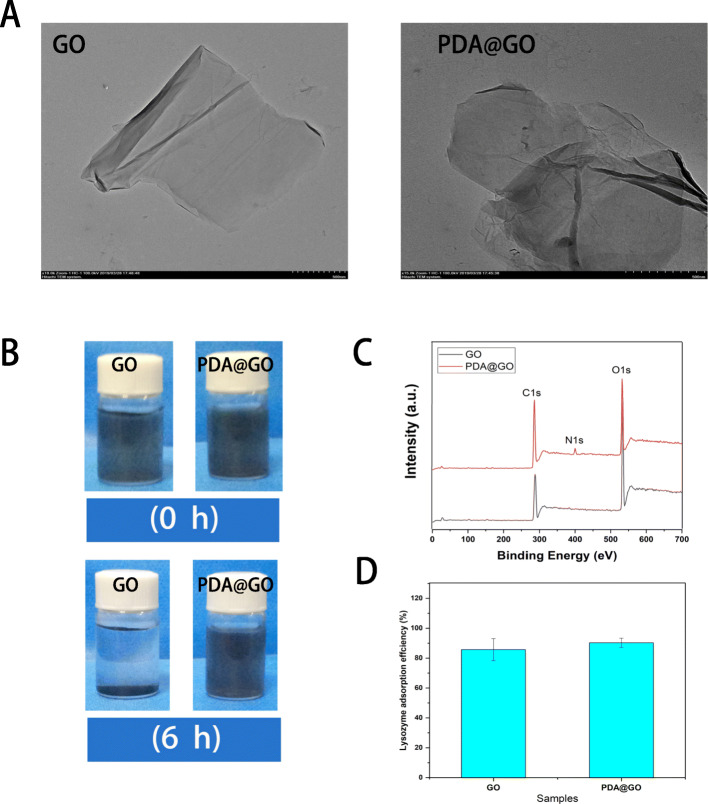
**a** TEM images of GO and PDA@GO, bar lengths are 500 nm; **b** The dispersion of GO (left) and GO@PDA (right) in ethanol after 6 h; **c** X-ray diffraction pattern of GO and PDA@GO; **d** Lysozyme adsorption effciency of GO and PDA@GO, *P* < 0.05, *n* = 3

The synthesis routes for ly-PDA@GO are shown in Scheme 1. Firstly, GO was immersed in a dopamine solutions, and dopamine can self-polymerize in an alkaline environment to form a stable PDA coating on the surface of GO. Then, PDA@GO was used be the drug carrier of lysozyme and immobilized on the surface of CS-Arg membrane through electro-static interactions. The XPS spectra and TEM images of the GO and PDA@GO were performed to determine the surface PDA coating. As shown in Fig. 1a, the GO presents a typical sheet-like structure with slight folds and small wrinkles. After PDA surface modification, the general structure of PDA@GO has not changed significantly, however, surface roughness is improved to some extent, and more wrinkled and folded regions could be clearly observed. This results indicated that dopamine is self-polymerized to create a coating on the surface of GO. Furthermore, the elements composition of GO and PDA@GO are also detected by XPS as shown in Fig. 1c. C and O elements were present in all GO and PDA@GO samples, but N element only existed in the PDA@GO and not in GO, which further indicated that successful combination of PDA and GO using in situ polymerization of dopamine. As shown in Fig. 1b, we used sedimentation tests to detect the dispersion of GO and PDA@GO in ethanol. In this test, GO has deposited into the bottom of the bottle after 6 h of storage while PDA@GO could maintain stable in ethanol for a longer time. After PDA surface modification, the dispersion stability of GO in aqueous solution was significantly improved, which make GO bind to water-soluble protein drugs more evenly and improve the loading efficiency of GO. Lastly, lysozyme was loaded onto the GO and PDA@GO surfaces via π-π stacking and charge interaction. The adsorptions of lysozyme on the GO and PDA@GO was examined (Fig. 1d). It is found that both GO and PDA@GO have very high adsorption capacity for lysozyme, and lysozyme adsorption rate is more than 85%. Previous studies have found that GO can strongly adsorbed protein with high selectivity such as lysozyme, which might due to the π-π interaction, hydrophobic interaction and electrostatic interaction of GO and protein [29]. Furthermore, the self-polymerized of dopamine cyclizes the amino (−NH_2_) group and leaves the hydroxyl (−OH) group, which releases the hydrogen cation and produces a negative charge on the surface of GO [37]. Therefore, PDA coating will generate more negative charge, so as to improve the adsorption capacity of GO to lysozyme through charge interaction. The above results showed that PDA@GO has excellent dispersion stability and can efficiently load protein drugs, so it is a very good drug carrier.

### Characterization of nanocomposite membrane

**Fig. 2:**
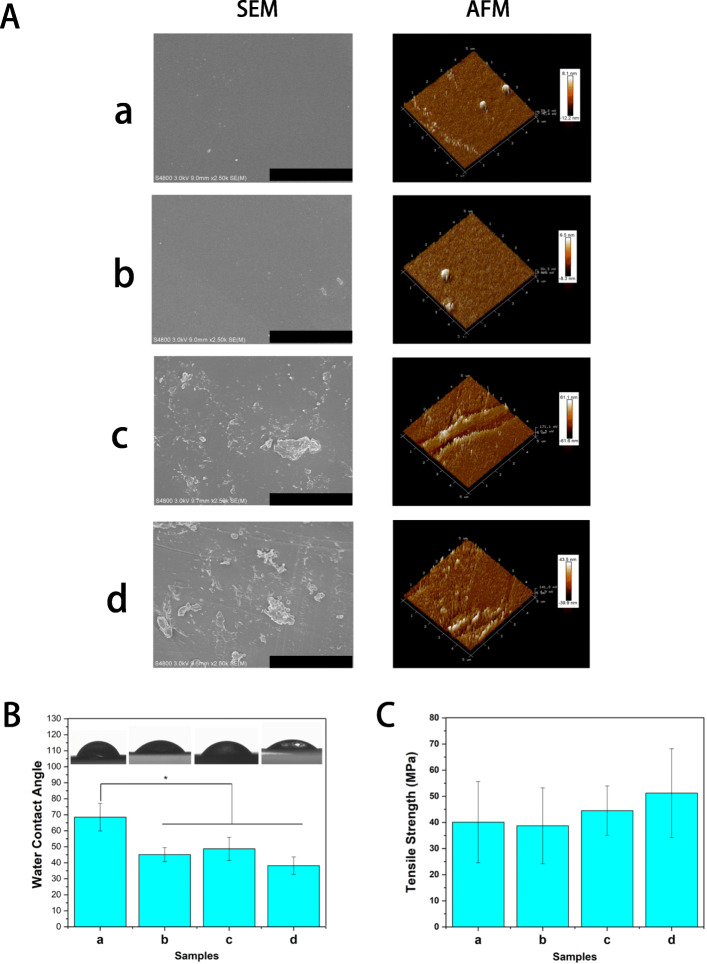
**A** SEM and AFM images of CS, CS-Arg, ly-GO/CS-Arg and ly-PDA@GO/CS-Arg nanocomposite membrane, bar lengths are 20 μm (SEM) and 5 × 5 μm (AFM). **B** Water contact angle and **C** tensile strength of CS (a), CS-Arg (b), ly-GO/CS-Arg (c) and ly-PDA@GO/CS-Arg (d) nanocomposite membrane, *P* < 0.05, *n* = 3

An ideal skin wound dressing should have good biocompatibility, tissue repair ability and antibacterial property. CS is a kind of natural alkaline polysaccharide, which has good biocompatibility and is widely used in tissue engineering. However, the tissue repair ability and antibacterial property of CS are limited, so bio-active factors are usually added to improve the repair effect. In this study, in order to improve the antibacterial property and biological activity of CS materials, CS membrane was modified by antibacterial peptide functionalizated GO and bio-active amino acid. The surface morphology of dressing materials plays an important role in cell adhesion proliferation and tissue engineering applications. The SEM image in Fig. 2A shows the surface morphology of different nanocomposite membrane (CS, CS-ArgCS-Arg, ly-GO/CS-Arg and ly-PDA@GO/CS-Arg). It was clear that the surface of pure CS membrane is relatively smooth and flat. A similar result was observed in the CS-Arg group, which demonstrated that the grafted Arg has little influence on the surface morphology of the CS membrane. With the addition of ly-GO and ly-PDA@GO, the surface roughness of the nanocomposite membrane is significantly increased and some agglomerated particles appears on the surface of membrane, which may be caused by the agglomeration of nanoparticles. Subsequently, we use AFM to further analyze the morphology and topological structure of different nanocomposite membrane. As show in Fig. 2A, the roughness root mean square (RMS) of CS and CS-Arg membrane were 10.15 nm and 7.4 nm, respectively. While the surface morphology of Ly-GO/Cs-Arg and ly-PDA@GO/CS-Arg films is rougher, and the surface roughness RMS is 61.35 nm and 41.7 nm, respectively. These results demonstrated that functionalized GO nanoparticles can enhance the surface topological structure of nanocomposite membranes, which may promote initial cellularization.

### Hydrophilicity and mechanical properties of nanocomposite membrane

The hydrophilicity of dressing material has significant influence on cell adhesion and proliferation. In general, highly hydrophilic surfaces can create a better cellular microenvironment for cell growth [38]. The wettability of different membrane can be determined by detecting the contact angle of water droplets on nanocomposite membrane surfaces [39]. As shown Fig. 2B, the contact angle of CS membranes was 68.5 ± 8.64, which decreased when L-Arg was grafted (45.1 ± 4.38). We believe that the hydrophilic enhancement of the nanocomposite membrane is due to the introduction of additional hydrophilic groups by Arg. Previous studies have found that Arg is rich in amines and carboxyl groups, which can not only react with CS materials, but also introduce more hydrophilic groups on the material surface [40]. When ly-GO was incorporated, these is no obvious change in the contact angle of nanocomposite membrane, due to GO also has good hydrophilic properties. Among all nanocomposite membrane, ly-PDA@GO/CS-Arg sample has the smallest contact angle, probably due to the hydrophilic nature of dopamine. Previous studies found that the cells have the best adhesion and growth behavior on the surface of materials with contact angle of 5 ~ 40, and such hydrophilic materials have better cellular compatibility [41]. The above results show that the hydrophilicity of CS materials can be improved by Arg and ly-PDA@GO, which is beneficial to its application in skin wound repair.

The mechanical properties of the dressing materials are also an important factor for wound healing. During wound healing process, the dressing materials should have the appropriate mechanical strength to adapt to the deformation caused by the wound contraction. In order to investigate the effects of ly-PDA@GO and l-Arg on the mechanical properties of CS membrane, we tested the tensile strength of CS, CS-Arg, ly-GO/CS-Arg and ly-PDA@GO/CS-Arg membrane. As shown Fig. 2C, the tensile strength of CS, CS-Arg, GO/CS-Arg and ly-PDA@GO/CS-Arg membrane were 40.1 ± 15.52, 38.7 ± 14.5, 44.5 ± 9.45 and 51.2 ± 17 MPa, respectively. Compared with pure CS and CS-Arg membrane, the tensile strength of ly-GO/CS-Arg membrane were slightly improved although there was no statistical difference, and the maximum tensile strength was achieved for sample of ly-PDA@GO/CS-Arg samples. Previous studies have found that there is a strong interfacial interaction between GO and chitosan, and the stress of the composite can be effectively transferred through the GO coating [42]. On the other hand, the PDA coating endow GO with stronger adhesion force, which provides a stronger interfacial interaction between GO and chitosan matrix. Furthermore, the more uniform distribution of PDA@GO on the CS-Arg membrane surface may also be one of the reasons for the enhanced mechanical strength. Based on the above results, the functionalized GO surface modification can improve the mechanical properties of chitosan materials, and this method can be carried out without damaging the material intrinsic structure.

### Antibacterial properties of nanocomposite membrane

**Fig. 3:**
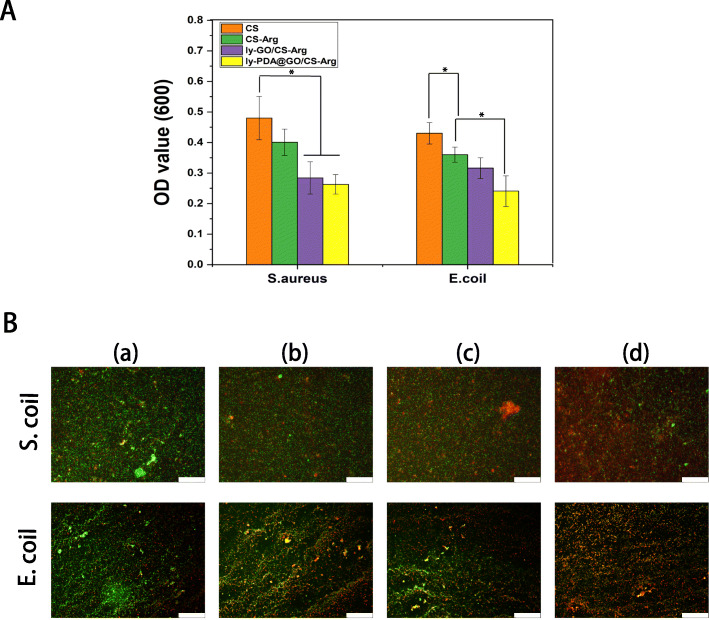
**A** Relatively antibacterial efficiency of different nanocomposite membrane, and **B** fluorescence micrographs of *S. aureus* and *E. coli* stained by PI & calcein after treatment with CS (a), CS-Arg (b), ly-GO/CS-Arg (c) and ly-PDA@GO/CS-Arg (d), Scale bar lengths are 100 μm, *P* < 0.05, *n* = 3

During the treatment of skin wounds, it is very important to prevent the bacterial infection of skin wound. One of the major challenges in treating wound infections is the overuse of antibiotics, which can lead to bacteria becoming resistant to antibiotics and eventually leading to severe chronic and recurrent infections. In this study, we fabricate a novel antibacterial dressing by the synergistic impact of functionalized GO and l-Arg. The antibacterial activity of different nanocomposites membrane was investigated using *E. coli* and *S. aureus*. As shown in Fig. 3, CS-Arg membrane against *S. aureus* and *E. coli* showed much better antibacterial activity than pure CS membrane, indicating that the surface grafted l-Arg can efficiently enhanced the antibacterial activity of CS materials. The surface charge density of positively charged CS materials can be increased by surface grafted l-arginine, so the antibacterial effect of CS-Arg is better than that of unmodified CS [43]. After adding ly-GO, the OD value of bacterial culture further decreased, which may be due to lysozyme was loaded into the nanocomposite membrane. Among all nanocomposite membrane, the ly-PDA@GO/CS-Arg nanocomposite membrane showed the highest bacterial inhibition. It is interesting that the growth of both *E. coli* and *S. aureus* was more inhibited by the ly-PDA@GO/CS-Arg nanocomposite membrane compared with ly-GO/CS-Arg nanocomposite membrane. Previous studies have shown that hydroxylated fullerenes can significantly inhibit lysozyme activity by binding to active sites [29]. GO is highly oxidized and has an SP2 carbon structure similar to hydroxylated fullerenes, which also significantly disrupts lysozyme activity. In the contrast, PDA@GO is less oxidizing and have little effect on enzyme activity. In order to better observe the antibacterial activity of different nanocomposite membranes, the bacteria on the surfaces of the different samples were stained with PI and calcein. Under fluorescence microscopy, dead bacteria showed red fluorescence, while living bacteria showed green fluorescence. All the samples, the green fluorescence of *E. coli* and *S. aureus* bacteria was strongest on the CS membrane. With the addition of Arg, GO and lysozyme, the red fluorescence region in the sample was gradually enhanced. Compared with other groups, the red fluorescence of ly-PDA@GO/CS-Arg membrane was the strongest, which verified that ly-PDA@GO/CS-Arg membrane had strong antibacterial activity.

**Fig. 4:**
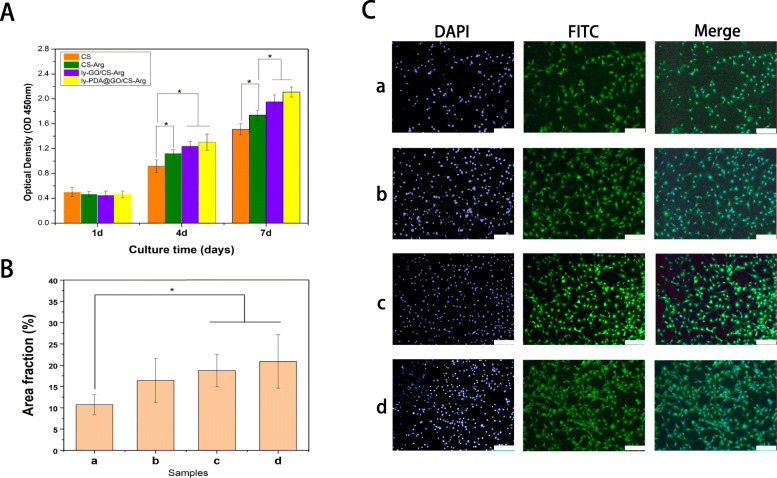
**A** Cell proliferation, **B** area fraction and **C** morphology of NH3T3 cells cultured on CS (a), CS-Arg (b), ly-GO/CS-Arg (c) and ly-PDA@GO/CS-Arg (d) nanocomposite membrane, Scale bar lengths are 200 μm, *P* < 0.05, *n* = 3

### Cell proliferation and adhesion

NH3T3 cells were used to systematically study the effects of different nanocomposite membrane on cell adhesion and proliferation. Cell proliferation on different membrane surfaces was detected using CCK-8 assay and FITC staining. As shown in Fig. 4A, the number of NH3T3 cells increased gradually with the culture time. At 4 and 7 days of culture, the number of cells in CS-Arg group was significantly higher than that in CS group, which proved the favorable effect of L-Arg modification on cell proliferation. After the addition of ly-GO and ly-PDA@GO, cell proliferation ability was further improved, especially at day 4 and 7. This might be because the addition of functionalized GO nanoparticles and Arg can improve the hydrophilicity and surface roughness of nanocomposite membrane, creating a better survival and adhesion conditions for the cells. Moreover, the abundant functional groups on GO surface can promote the interaction between membrane materials and cells. In 4 and 7 days of culture, the highest number of cells grew in the ly-PDA@GO/CS-Arg group. This may be due to the presence of PDA on the membrane surface. PDA contain a large number of active functional groups, such as catechol groups, which can generate a cation-π or π-π interactions with the cell membrane and promote cell adhesion [25, 44].

To better observe the effect of the different composite membrane on cell adhesion, the morphology of the NH3T3 grown on different membrane at 4 d was observed using fluorescence microscope by cytoskeleton (green) and nuclei (blue) staining. As show in Fig. 4 B,C, among all nanocomposite membrane, the number of cell on CS membrane was least, and cell adhered on CS membrane did not spread completely and showed a round or spindle morphology. With the addition of Arg, the number of cells on the CS-Arg membrane surface increased significantly, and the cells on membrane surface formed a distinct pseudopod structure, and the length of the pseudopod increased. Compare with CS-Arg group, the number of cells on ly-GO/CS-Arg and ly-PDA@GO/CS-Arg membrane was higher, and the cells adhered on the membrane modifed by ly-GO and ly-PDA@GO, especially in the ly-PDA@GO/CS-Arg group, were fully spread, the cytoskeleton was clear. This cell morphology showed a consistent result with CCK-8 assay that the ly-PDA@GO/CS-Arg nanocomposite membrane was safe enough for mammalian cells and have excellent biocompatibility. More importantly, Arg and functionalized-GO surface modification can not only improve the antimicrobial activity of CS, but also make the composites perform more excellent tissue repair ability.

### Evaluation of rat epidermal injury repair

**Fig. 5:**
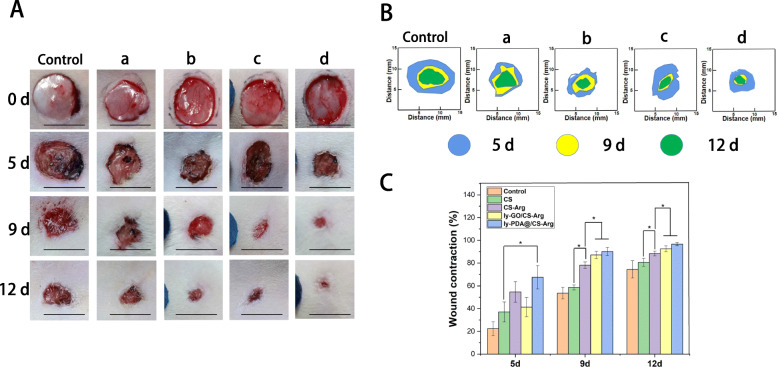
**A** Photographs of the wound closure and **B** the schematic diagram of wounds closure on different time point; **C** Quantitative statistical analysis of wounds closure for CS (a), CS-Arg (b), ly-GO/CS-Arg (c) and ly-PDA@GO/CS-Arg (d) tretment, *P* < 0.05, *n* = 3

Skin is the main barrier to protect the body from external stimulation and injury, and the skin tissue regeneration is a complex multi-factor process, which undergoes different stages of healing cascade. In order to analyze the in situ applicability and the effect of different nanocomposite membrane on skin wounds, the skin wounds of SD rats are treated with CS, CS-Arg, ly-GO/CS-Arg and ly-PDA@GO/CS-Arg membrane. The nanocomposite membrane was changed every 2 days, and the wound was photographed. Meanwhile, the area of the wound was measured using the software Image J. The photographic images and closure rate of wounds after treatment on days 0, 5, 9, and 12 are shown in Fig. 5A. With the prolongation of time, the size of the wound tended to decrease in each group. Among all groups, the control group without any treatment showed the slowest rate of wound healing. Furthermore, it can be found that the wound closure rate of the CS-Arg group is higher than that of the CS group, which demonstrated that the addition of Arg can improve the repair efficiency of the nanocomposite membrane. According to reports, the L-Arg have a positive effect on normalize or enhance wound healing in humans; they also has profound effects on keratinocytes performance during the process of healing [45, 46]. Among all group, the ly-PDA@GO/CS-Arg and ly-GO/CS-Arg treatment shows the fastest reduction in wound area and the best closure rate. As shown in Fig. 5B and C, the wound closure rate of the ly-PDA@GO/CS-Arg and Ly-GO/Cs-Arg group was much higher than that of the other groups after 9 days of treatment, reaching 87.11 and 90.1%, respectively. The wound is almost completely closure in the the ly-PDA@GO/CS-Arg and Ly-GO/Cs-Arg group after treatment for 12 days. We speculate that nanoparticles loaded with antimicrobial peptides can effectively improve the antimicrobial properties of the nanocomposite membrane and inhibit infections caused by the pathogens, which can creates a more ideal healing environment for the wound. Moreover, previous studies have found that GO had a good biocompatibility and could effectively promote tissue regeneration [47, 48]. The abundant functional groups of PDA@GO could also interact with cells and improve cell viability. Thus, in the mouse wound healing model, the ly-PDA@GO/CS-Arg nanocomposite membranes could significantly accelerated wound healing and shortened wound closure time.

### Histological analysis of skin repair

**Fig. 6:**
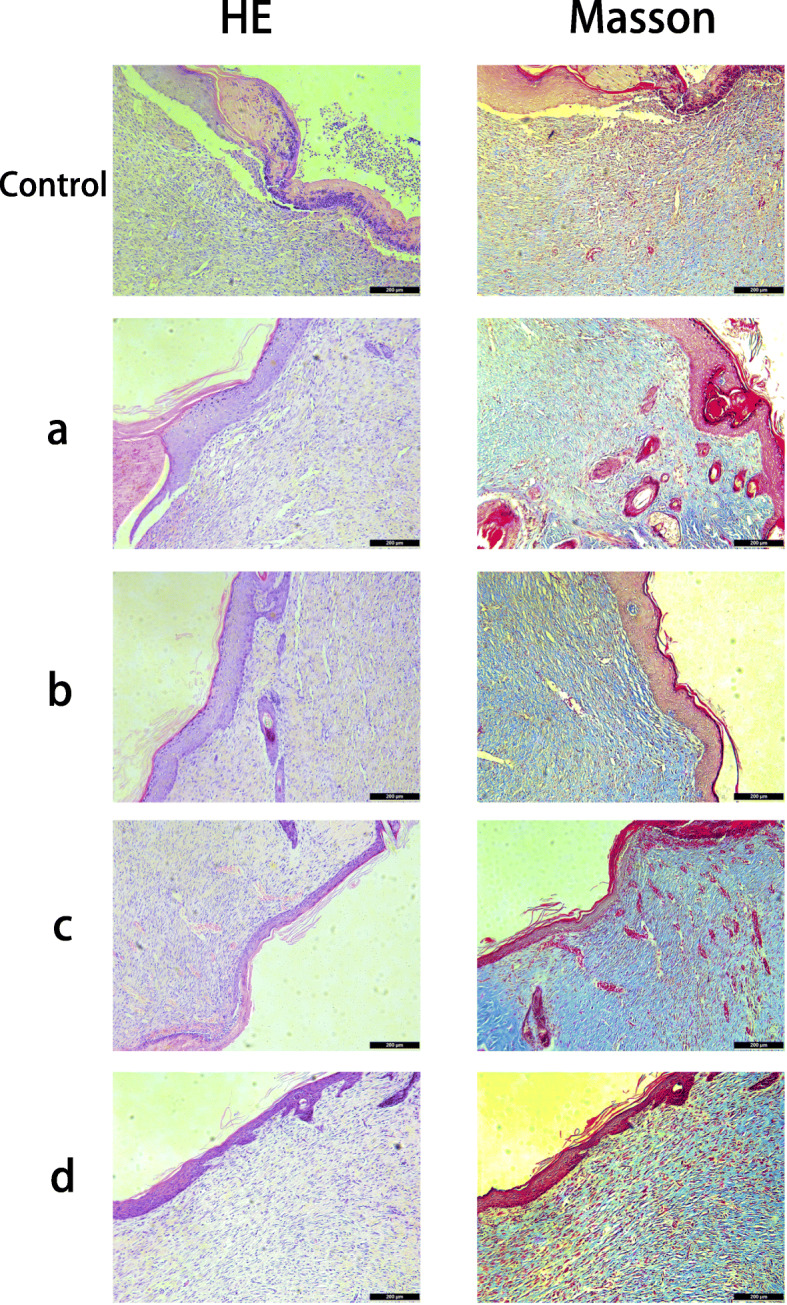
Histological appearance of wounds harvested on days 12 of each group, CS (**a**), CS-Arg (**b**), ly-GO/CS-Arg (**c**) and ly-PDA@GO/CS-Arg (**d**), scale bar lengths are 200 μm

After evaluating the effect of different nanocomposite membranes on increasing the wound healing rate, we further analyzed the repair effects of different materials at the histological level. Histological analysis was performed on the H&E-stained and Masson-stained tissue samples to gain a deeper understanding of the effect of different nanocomposite membranes on wound healing. As shown in Fig. 6, after CS-Arg, ly-GO/CS-Arg and ly-PDA@GO/CS-Arg membrane treatment, the wound was largely healed, the new tissue was denser and fewer inflammatory cells had infiltrated as compared with results in the CS and control group. Furthermore, the epidermal thickness of skin samples in each group was significantly different. The the control group without any treatment had a significantly thicker epidermis than those treated with nanocomposite membranes. With the addition of Arg, ly-GO and ly-PDA@GO, the epidermal thickness of samples further decreased. Among all groups, ly-PDA@GO/CS-Arg group had the thinnest epidermal thickness, which was close to normal skin tissue. At the beginning of epidermal healing, the epidermis gradually thickens to greater than normal thickness as cells proliferate. As healing continues, epidermal thickness returns to normal during maturation. Thus, the above results suggested that the wound healing degree of ly-PDA@GO/cs-Arg group was higher than that of other groups. The result of collagen deposition in each group by modified Masson’s trichrome staining on day 12 is shown in Fig. 6. The result show that the CS-Arg, ly-GO/CS-Arg and ly-PDA@GO/CS-Arg groups had a darker blue color than the CS and control group, suggesting that those group have a higher collagen deposition in the wound site. Furthermore, collagen fibers in the dermal layer of the control and CS groups were arranged irregularly and sparser, while collagen had a regular arrangement in the CS-Arg, ly-GO/CS-Arg and ly-PDA@GO/CS-Arg treated group, which further demonstrated the maturity of the regenerated tissue.

**Fig. 7:**
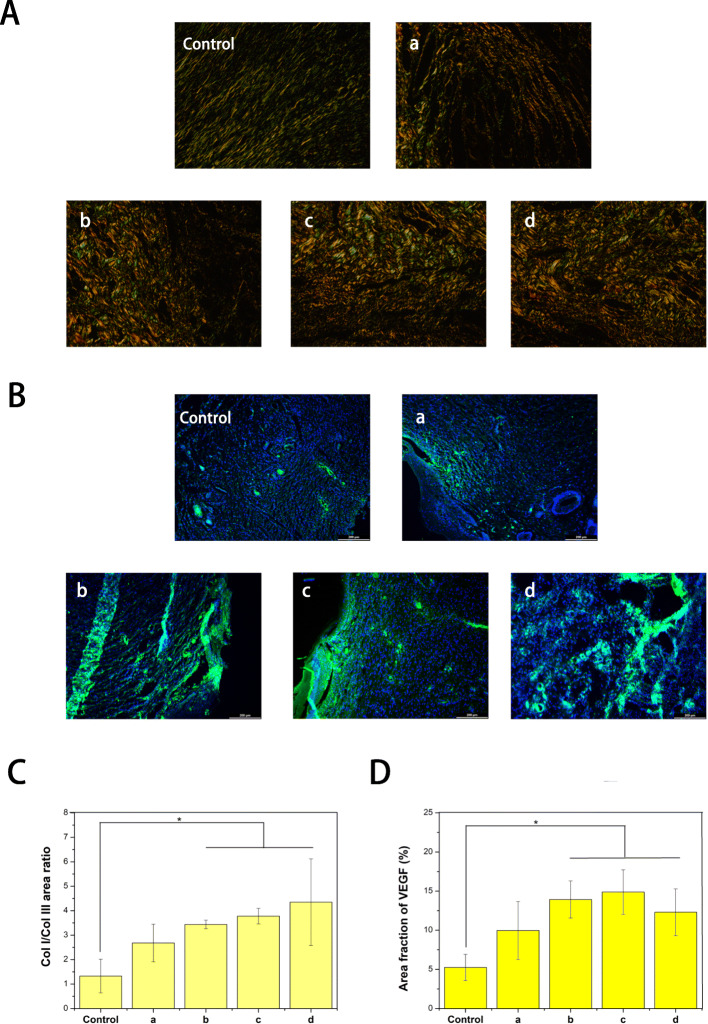
**A** Sirius red staining and **C** the COL I/COL III area ratio in the wound-healing region at 12 days after CS (a), CS-Arg (b), ly-GO/CS-Arg (c) and ly-PDA@GO/CS-Arg (d) treatments, Magnification, 200×, *P* < 0.05 (*n* = 3). **B** Immunofluorescence images and **D** the quantitative statistical analysis of VEGF relative area percentage in the wound-healing region at 12 days after CS (a), CS-Arg (b), ly-GO/CS-Arg (c) and ly-PDA@GO/CS-Arg (d) treatments, scale bar lengths are 200 μm, *P* < 0.05 (*n* = 3)

Collagen is the main component of skin tissue, and there are two types of collagen involved in the process of skin wound healing, collagen type I (COL-I) and collagen type III (COL-III). At the early phase of wound healing, the new tissue is rich in thin and loose COL-III, while the expression of COL-I is weak [49]. As the tissue recovery process advances, COL-III is gradually replaced by COL-I to increase the tensile strength of the scar. Therefore, the ratio of COL-I to COL-III can be used as an indicator to evaluate the degree of wound healing. Sirius red staining was used to analyze the type of collagen in wound (COL-I show orange to red color and COL-III show green). As shown in Fig. 7A, the vast majority of wound collagen in all groups was COL-I. However, in the control group, the proportion of COL-III was higher, indicating that the wound was still healing. It was found that COL-I expression was increased in the CS-Arg samples compared with that of the CS groups. Actually, different reports in the literature have described that l-arginine can improve collagen deposition and the wound tensile strength [50]. Therefore, l-Arg can improves wound healing by increasing collagen synthesis. When ly-GO or ly-PDA@GO was added, the COL-I content in the skin wound samples further increased and the fluorescence intensity increased, indicating that ly-GO or ly-PDA@GO greatly promoted the rate of wound healing. Antimicrobial peptides-functionalized GO nanoparticles have a strong inhibitory effect on the growth of pathogens, which effectively improves the antimicrobial properties of nanocomposite membrane and provides a prerequisite for rapid wound healing. More importantly, GO nanoparticles can improve the physical properties of nanocomposite membrane, including mechanical strength, hydrophilicity and protein adsorption. All the above factors can effectively improved the tissue repair ability of the nanocomposite membrane. Furthermore, we found that the ly-PDA@GO/CS-Arg group had the highest proportion of type I/III collagen among all groups, indicating that the ly-PDA@GO/CS-Arg group had been in the remodeling stage and had the highest efficiency of wound repair. Finally, we also detected the expression of VEGF in wound tissues. As shown in Fig. 7B, green fluorescence in the CS-Arg, ly-GO/CS-Arg and ly-PDA@GO/CS-Arg groups was significantly higher than that in the other groups on the 12th day, which confirmed the mechanism of nanocomposite membrane achieving better repair effect by promoting angiogenesis.

Chitosan is a well-known wound repair material, which has excellent biocompatibility and degradability, and can enhance fibroblast migration and collagen deposition [9, 51]. Furthermore, chitosan is cheap, easy to be modified and processed, and can be used in the development of healthcare products. However, there are still some drawbacks when chitosan is used for wound repair, such as low absorbability, easy degradation, poor mechanical strength, low antibacterial activity. In order to overcome the above drawbacks, many approaches have been adopted to incorporate functional materials into the chitosan materials to enhance its antibacterial activity and tissue repair ability [52, 53]. In recent years, adding carbon-based nanomaterials such as graphene and carbon nanotubes to enhance the biological properties of chitosan has become a research hotspot [54, 55]. Furthermore, GO can be used as an excellent carrier of bio-active factors due to the existence of abundant functional groups on GO surface, such as epoxy (C-O-C), hydroxyl (OH) or carboxyl (COOH), expanding the biomedical applications of graphene-based materials. La et al. found that GO could be used for BMP-2 delivery, which greatly improve the use efficiency of growth factors [56]. Yang et al. developed a novel drug carrier based on functionalized GO, which can precisely delivers doxorubicin to the cancer cells that overexpress the CD44 receptor [57]. However, there are few studies on GO as a drug carrier that be applied to modify tissue repair material at present. In this study, we used PDA@GO as the drug carrier of lysozyme, and combined ly-PDA@GO with CS-Arg membrane by electrostatic interaction. The results showed that PDA@GO had good water dispersion and affinity for lysozyme, and had no negative effect on the activity of lysozyme. Thus, PDA@GO can be used as an excellent drug-carrier of antimicrobial peptides. By testing the influence of PDA@GO on the mechanical strength, hydrophilicity and antibacterial properties of the nanocomposite membrane, we found that ly-PDA@GO/CS-Arg nanocomposite membrane exhibited superior properties among the other membrane. The cell experiments showed that the biocompatibility and tissue repair ability of CS material were further improved by surface modification with ly-PDA@GO and Arg. Compared with other nanocomposite membranes, more cell number and higher cell proliferation activity were found in ly-PDA@GO/CS-Arg nanocomposite membrane, which may be attributed to Arg and ly-PDA@GO improve the surface hydrophilicity and roughness of the material, and provide more cell binding sites for the cells. On the other hand, the antibacterial peptides and abundant functional group on PDA@GO can also improve cell activity and inhibit the growth of bacteria. More importantly, the results of our study in rats showed that the surface modification of Arg and ly-PDA@GO had a certain synergistic effect on wound healing, and the ly-PDA@GO/CS-Arg nanocomposite membrane could effectively accelerate wound healing, accelerate epidermal remodeling, and promote the formation of collagen deposition. Therefore, our study not only provides a potential biomaterial for epidermal wound repair, but also contributes to a better understanding of the biological applications of graphene-based nanomaterials.

## Conclusions

In the this study, antimicrobial peptides (lysozyme) onto PDA@GO was successfully loaded, and the functionalized GO was used to cover CS-Arg nanocomposite membrane through elec-trostatic interactions. The prepared nanocomposite membrane showed a good biocompatibility, hydrophilicity and antibacterial properties. On the other hand, the good biocompatibility of these nanocomposite membrane has also been verified by cell proliferation and adhesion assay. Finally, in the mouse full-thickness defect repair model, the better wound repair effect of prepared nanocomposite membrane in skin wound were demonstrated. Taken together, we have innovatively modified GO and combined it with CS-Arg materials for potential applications in skin tissue engineering.

## Supplementary Information

**Additional file 1: Fig. S1.** HE images of wound edge in different group. CS (a), CS-Arg (b), ly-GO/CS-Arg (c) and ly-PDA@GO/CS-Arg (d), scale bar lengths are 500 μm

## Data Availability

The raw data used and/or analyzed during the current study are available from the corresponding author on reasonable request.

## Abbreviations

CS: Chitosan
Arg: L-arginine
GO: Graphene oxide
ly: Lysozyme
PDA: Poly-dopamine
NO: Nitric oxide
CCK-8: Cell Counting Kit-8
SEM: Scanning electron microscope
AFM: Multimode scanning probe atomic force microscope
