# Supplementary material for: Enhanced wound repair ability of arginine-chitosan nanocomposite membrane through the antimicrobial peptides-loaded polydopamine-modified graphene oxide
Source: J Biol Eng. 2021 May 22;15:17. doi: 10.1186/s13036-021-00268-3 (PMC8141257; doi:10.1186/s13036-021-00268-3)
Supplement: Supplementary file 1 — Additional file 1: Fig. S1. HE images of wound edge in different group. CS (a), CS-Arg (b), ly-GO/CS-Arg (c) and ly-PDA@GO/CS-Arg (d), scale bar lengths are 500 μm [file 13036_2021_268_MOESM1_ESM.doc]

**Support information for**

**Enhanced wound repair ability of arginine-chitosan nanocomposite membrane through the antimicrobial peptides-loaded polydopamine-modified graphene oxide**

Chuan Fu^1^, Zhiping Qi^1^, Chengliang Zhao^2^, Weijian Kong^1^, Hongru Li^1^, Wenlai Guo^2*^, and Xiaoyu Yang^1*^

^1^ Department of Orthopedic Surgery, the Second Hospital of Jilin University, Changchun 130021, China

^2^ Department of Spinal Surgery, the Affiliated Hospital of Qingdao University, Qingdao 266000, China

Current Address: Department of Orthopedic Surgery, the Second Hospital of Jilin University, Changchun 130021, China

*Corresponding Author

E-mail: yangxiaoy@jlu.edu.cn (Xiaoyu Yang);

E-mail: guowl16@mails.jlu.edu.cn (Wenlai Guo);

**Contents:**

Figure S1

**Supplementary Figures**


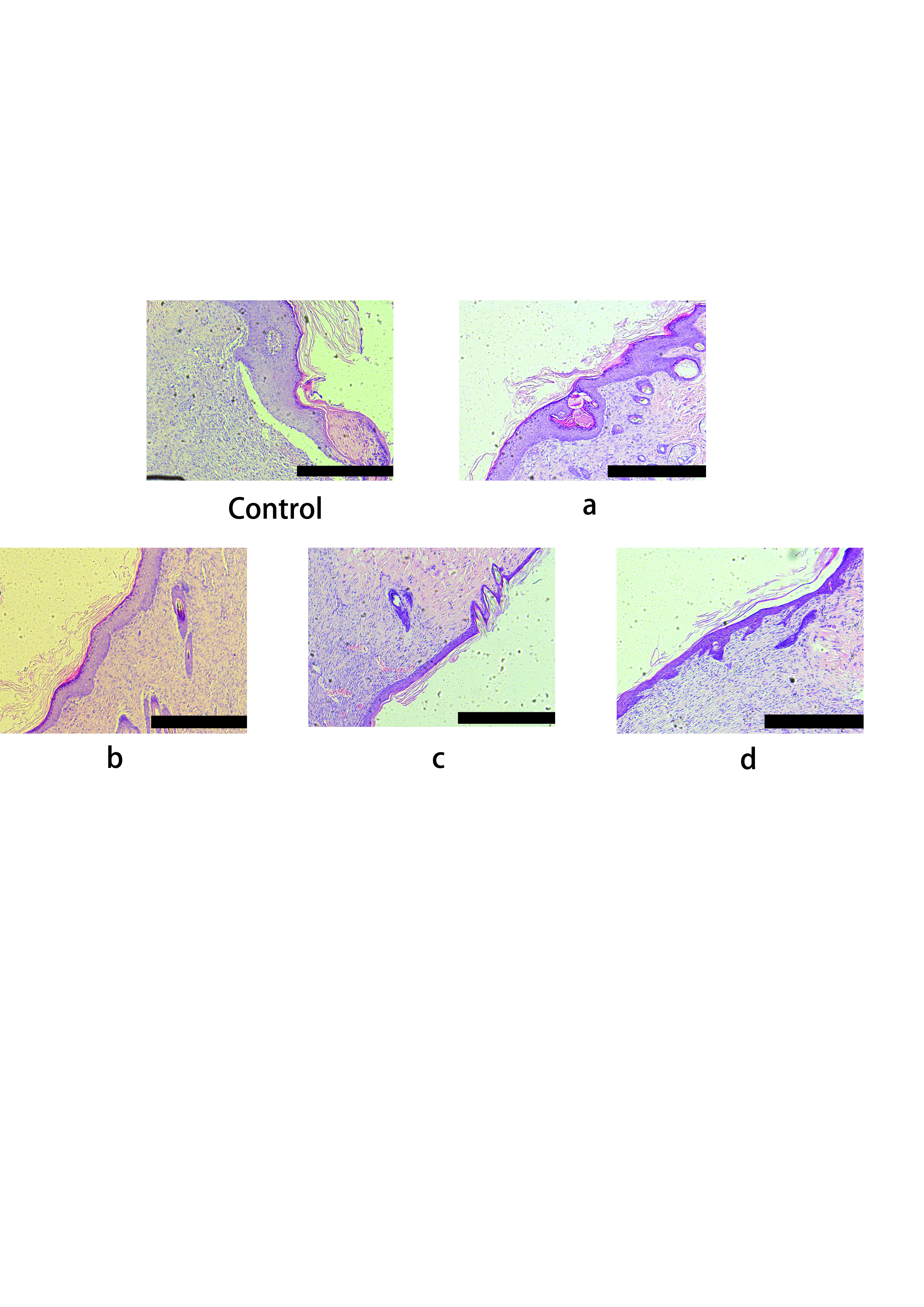


**Fig. S1** HE images of wound edge in different group. CS (a), CS-Arg (b), ly-GO/CS-Arg (c) and ly-PDA@GO/CS-Arg (d), scale bar lengths are 500 μm.
